# *Mucor thermorhizoides*—A New Species from Post-mining Site in Sudety Mountains (Poland)

**DOI:** 10.1007/s00284-024-03708-7

**Published:** 2024-06-01

**Authors:** Beniamin M. Abramczyk, Dorota G. Wiktorowicz, Alicja Okrasińska, Julia Z. Pawłowska

**Affiliations:** 1 Biology of Microorganisms Students’ Society, Faculty of Biology, University of Warsaw, ul. Miecznikowa 1, 02-096 Warsaw, Poland; 2 Institute of Evolutionary Biology, Faculty of Biology, Biological and Chemical Research Centre, University of Warsaw, ul. Żwirki i Wigury 101, 02-089 Warsaw, Poland

## Abstract

*Mucor* representatives are mostly rapidly growing cosmopolitan soil saprotrophs of early diverged *Mucoromycotina* subphylum. Although this is the most speciose genus within the group, some lineages are still understudied. In this study, new species of *Mucor* was isolated from the post-mining area in southwestern Poland, where soil chemical composition analysis revealed high concentration of hydrocarbons and heavy metals. Phylogenetic analysis based on multigene phylogeny showed that the new isolate clusters distinctly from other *Mucor* species as a sister group to *Mucor microsporus.* New species *Mucor thermorhizoides* Abramczyk (*Mucorales, Mucoromycota*) is characterized by the extensive rhizoid production in elevated temperatures and formation of two layers of sporangiophores. It also significantly differs from *M. microsporus* in the shape of spores and the size of sporangia. *M. thermorhizoides* was shown to be able to grow in oligotrophic conditions at low temperatures. Together with *M. microsporus* they represent understudied and highly variable lineage of the *Mucor* genus.

**Supplementary Information:**

The online version contains supplementary material available at 10.1007/s00284-024-03708-7.

## Introduction

*Mucor* is the most speciose genus within *Mucoromycotina,* including over 100 accepted species [1]*.* The type species of the genus *Mucor* is *Mucor mucedo* which Linneus described in the second volume of Species Plantarum [2]. The generic name *Mucor* based on an earlier description by Pier Antonio Micheli was also proposed in the same publication. The genus was also described by Fresenius [3] almost a hundred years later in Beiträge zur Mykologie. In 1986, Paul Kirk proposed to conserve *Mucor* Fres. over *Mucor* Micheli ex L.: Fr., defining the current understanding of the genus [4]. Traditionally, the taxonomy within the genus was based on morphology and mating experiments (e.g., [5]). Due to the large variability and the high number of species, Zycha [6] proposed to divide the *Mucor* genus into sections based on the shape and height of sporangiospores, and the colour of the culture. This system was further developed by Hesseltine [7] who delimited nine sections within the genus *Mucor:* Flavus, Fragilis, Genevensis, Hiemalis, Macromucor, Mucedo, Racemosus, Ramannianus, and Sphaerosporus. According to the genus description [3], *Mucor* species produce non-apophysate, globose sporangia on simple or branched sporangiophores arising directly from the substrate, without stolons or rhizoids.

In recent decades, molecular tools have become standard in fungal taxonomy, resulting in several revisions of *Mucor* (e.g., [8–10]) proving that some of the above-mentioned morphological characters are not taxonomically informative [11]. Moreover, the genus in its current sense still remains polyphyletic [10]. Walther et al. [9] showed that only a few morphologically recognized groups are confirmed by the phylogenies (i.e., *M. mucedo* group, *M. flavus* group, *M. hiemalis* group, *M. racemosus* group and *M. amphibiorum* group). On the other hand, molecular studies revealed large diversity amongst *Mucor* representatives [9], several new species are described within the genus each year (e.g., [11]) and many more dark taxa—known only from environmental DNA (eDNA) sequencing—are reported [12].

The genus *Mucor* groups mainly rapidly growing soil saprotrophs [8, 9, 13]. Although known as cosmopolitan fungi, for the majority of species, ecological roles and geographical distributions are poorly studied [11]. More than 20,500 occurrences recorded in GBIF prove that *Mucor* representatives are present on all continents including Antarctica (GBIF.org accessed 5.12.2022) [14]. According to eDNA amplicon-based biodiversity studies, *Mucor* is detected mostly in the soil, and more than 70% of sequences assigned to *Mucor* originated from forests [12]. Although representatives of this group were also recently indicated as underrepresented both in terms of species and records in forest biodiversity research [15].

One of such understudied, dark *Mucor* taxon is a clade defined in UNITE as species hypothesis SH1102029.09FU [16]. It is composed of 4 full-length ITS (nuc rDNA ITS1-5.8S-ITS2) sequences. One of them originated from an unidentified *Mucor* isolated from archaeological wooden artefacts in Western Greenland [17] and three others represent eDNA from soil samples also from Greenland. Although the representative of SH1102029.09FU has been already isolated by Pedersen [17], it was never formally described. Such description could contribute to solving the problem of lack of phenotypic and physiological traits for some fungi known only from eDNA data, which was highlighted by Hibbett et al. [18].

Whilst studying the diversity of soil *Mucoromycota* fungi in the post-mining area of south-western Poland, we isolated a *Mucor* strain that matched SH1102029.09FU and differed morphologically from other known species. Based on phylogenetic analysis of ITS, LSU (fragment of nuc 28S rDNA approximately 700 bp), *MCM7* (fragment of Minichromosome Maintenance Complex Component 7 gene), *RPB1* (largest subunit of RNA polymerase II), *TSR* (20S rRNA accumulation protein encoding gene) and *CFS* (fragment of a gene predicted to encode for a cyclopropane-fatty-acyl-phospholipid synthase) genetic markers, as well as morphological characterization, we thus propose to describe our isolate as a new species—*Mucor thermorhizoides* Abramczyk sp. nov.

## Materials and Methods

### Sampling Site and Isolation

**Fig. 1:**
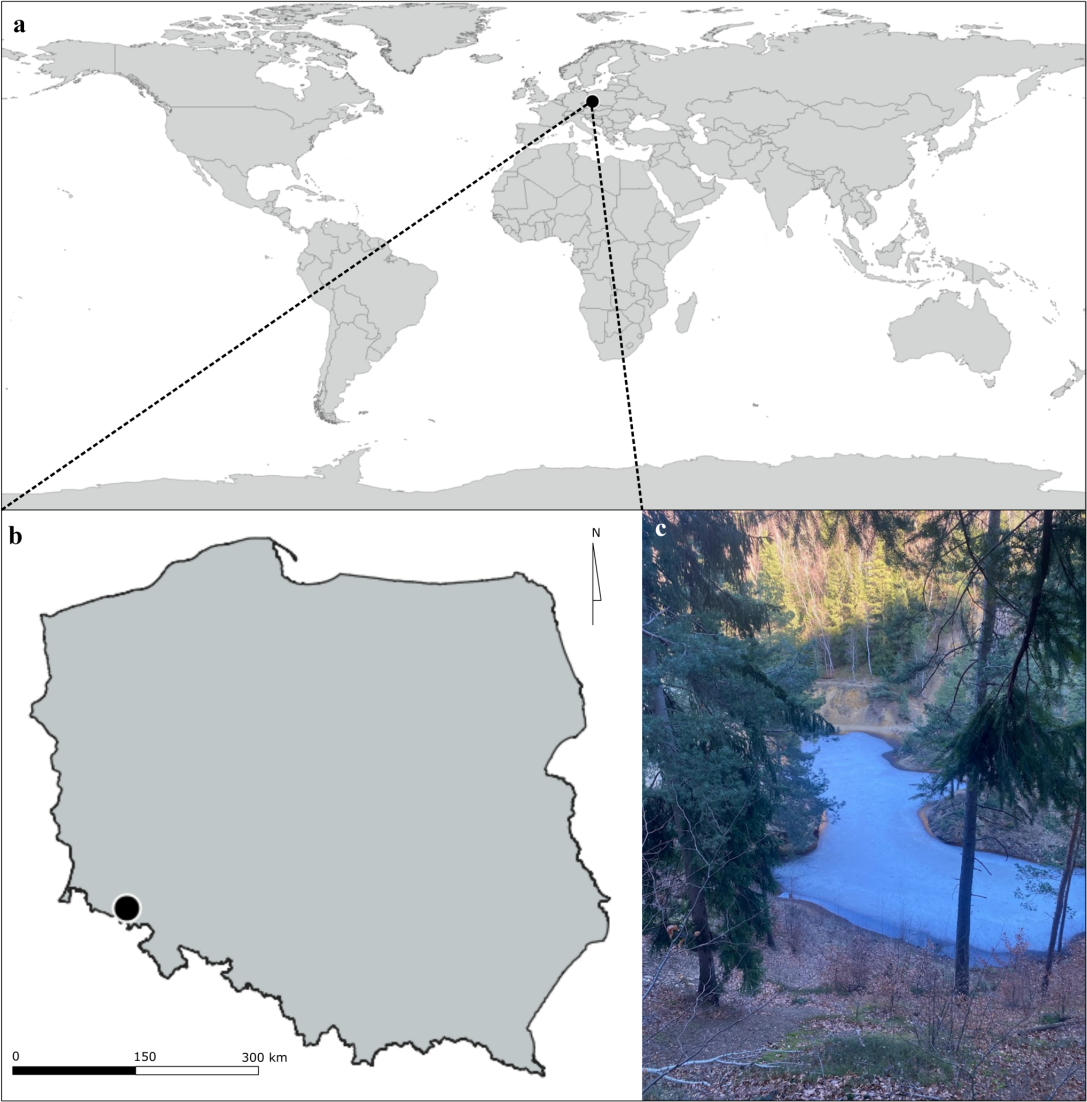
Characteristics of sampling site **a** localization on a global scale; **b** localization in Poland; **c** general view of sampling habitat

Soil sample was taken from a path between “Purple Lake” (Purpurowe Jeziorko) and “Blue Lake” (Niebieskie Jeziorko) in a protected nature area of a former pyrite mine in Rudawy Janowickie (Wieściszowice, Marciszów, Lower Silesia, Poland; coordinates: 50°49.512′ N, 15°58.332′ E, WGS84) on 25.06.2018 (Fig. 1). Sampling location maps were created in qgis 3.28.3 [19] and compiled into one figure with a photograph in inkscape (inkscape.org).

The vegetation in the sampling area is several decades-old spruce forest. Soil pH at the moment of sampling was 5.0, and its temperature equalled 2 °C. After 2 years of storage at − 20 °C, the soil was used for further analyses.

Culturable fungi were isolated from 2.5 g of soil per Petri dish using the Warcup method [20] on Water Agar (WA) medium (BTL Sp. z.o.o., Łódź, Poland). Plates were incubated at room temperature (~ 23 °C) until fungal growth was visible under a stereo microscope. Single sporangia were transferred with a preparation needle onto Malt Extract Agar (MEA) medium (BTL Sp. zo.o., Łódź, Poland) to obtain axenic cultures. After one week of incubation at 23 °C, microscopic observation and further steps were performed.

One *Mucor* isolate (two clones) that matched SH1102029.09FU and differed morphologically from other known species was isolated from this soil sample.

### Soil Chemical Composition

Inductively coupled plasma optical emission spectrometry was used to determine selected heavy metals concentrations, i.e., arsenic (As), aluminium (Al), cadmium (Cd), cobalt (Co), chromium (Cr), copper (Cu), iron (Fe), molybdenum (Mo), manganese (Mn), nickel (Ni), lead (Pb), vanadium (V), and zinc (Zn). Mercury (Hg) concentration was measured with the use of atomic absorption spectroscopy with mercury analyzer AMA 254. Concentrations of total petroleum hydrocarbons (C6–C12); mineral oils (C12–C35); (ethyl)benzene, toluene, three xylene isomers, styrene (BTEX); polycyclic aromatic hydrocarbons (PAH) were determined using GC–MS (gas chromatography with a mass spectrometer) and GC-FID (gas chromatography with flame ionization detector). Wessling Company performed the above analyses.

### Morphological Observations

Culture characteristics were studied on malt extract agar (MEA). Macroscopic features were viewed and photographed using 7-day-old cultures incubated at 23 °C. Colony colours were determined with the Ridgway colour standards [21]. Morphological analyses were made under a Nikon Eclipse E600 light microscope at a magnification of × 400 and × 600. The microscopic structures were measured and photographed using NIS-elements br 3.1 software (Nikon). Photographs were compiled in inkscape (inkscape.org).

Yeast-growth test was performed in YPG (Yeast-Peptone-Glucose) broth medium after 3 days of growth according to the protocol described by Wagner et al. [8].

### Growth Temperature

The colony growth test was performed at 4, 23, 25, and 30 °C on MEA (Malt Extract Agar), PDA (Potato Dextrose Agar), SDA (Sabouraud Dextrose Agar), and WA (Water Agar) media in triplicate (all produced by BTL Sp. zo.o., Łódź, Poland). Three-day-old fungal cultures were placed in one of the temperatures mentioned above. The size of the colonies was measured at the beginning and after 7 days of incubation.

As the maximum growth temperature is a diagnostic character for some mucoralean fungi [22], the test proposed by Wagner et al. [8] was performed in five different temperatures (room temperature—about 23, 25, 26, 27, and 28 °C). For each temperature the test was performed in five replicates. Briefly, the fungus was grown for 3 days on MEA medium at room temperature (~ 23 °C) on 90 mm Petri dishes. Then, the border of each colony was drawn on a plate with a marker pen and plates were further incubated at one of the different temperatures for the next 7 days. The colony's size at each temperature was measured at the beginning, and after 3 and 7 days of incubation.

All diagrams were plotted using R [23].

### DNA Isolation, Amplification, and Sequencing

Total genomic DNA was extracted from fungal cultures using an ExtractMe Genomic DNA Kit (Blirt S.A., Gdańsk, Poland), according to the manufacturer’s instructions. 6 molecular markers were amplified: ITS using primer pair ITS1f and ITS4 [24], LSU using primer pair NL1 and NL4 [25], *MCM7* using primer pair Mcm7-709 and Mcm7-1348rev, *CFS* using primer pair CFS-f1 and CFS-r1, *RPB1* using primer pair RPB1-f1 and RPB1-R3096, and *TSR* using primer pair TSR1-f2 and Tsr1-r2 [8]. PCR mix for ITS, LSU, *MCM7* and *CFS* contained: 2 × PCR TaqNova-RED PCR Master Mix (Blirt S.A., Gdańsk, Poland) (10 μL), forward and reverse primers in 10 μM concentration (1.5 μL each), water (4 μL) and obtained DNA solution (3 μL). PCR mix for *RPB1* and *TSR* amplification contained: 10 × CoralLoad PCR Buffer (2 μL), 10 mM dNTP solution (0.4 μL), Taq DNA Polymerase (0.1 μL), water (14 μL), 25 mM MgCl_2_ solution (0.5 μL), Q-Solution (1 μL) (QIAGEN, Venlo, Netherlands), forward and reverse primers in 10 μM concentration (0.5 μL each), 3 ng of obtained genomic DNA (1 μL). PCR reactions were performed using BIO-RAD T100 Thermal Cycler. The following amplification programmes were used: for ITS marker: 4 min in 95 °C for initial denaturation, 34 cycles of 30 s in 95 °C, 30 s in 52 °C for annealing, 1 min in 72 °C, and 10 min in 72 °C for final elongation; for LSU marker: 5 min in 94 °C for initial denaturation, 35 cycles of 30 s in 95 °C, 1 min in 55 °C for annealing, 1 min in 72 °C, and 5 min in 72 °C; for *MCM7* marker: 5 min in 95 °C for initial denaturation, 30 cycles of 30 s in 95 °C, 30 s in 54 °C for annealing, 1 min in 72 °C, and 10 min in 72 °C for final elongation; for *CFS* marker: 5 min in 95 °C for initial denaturation, 30 cycles of 30 s in 95 °C, 30 s in 55 °C for annealing, 1 min in 72 °C, and 10 min in 72 °C for final elongation; and for *RPB1* and *TSR* markers: 5 min in 95 °C for initial denaturation, 60 cycles of 30 s in 95 °C, 1 min in 53 °C for annealing, 1.5 min in 72 °C, and 5 min in 72 °C. The PCR products were visualized on 1% agarose gels stained with Midori Green Advance (Nippon Genetics Europe). PCR products were purified using Extract Me DNA Clean-Up and Gel Out kit (Blirt S.A., Gdańsk, Poland) according to the manufacturer’s protocol. ITS, *MCM7* and *CFS* were sequenced using the ABI PRISM BigDye Terminator Cycle Sequencing Ready Reaction Kit 3.1 (Applied Biosystems, Warrington, UK) with the same primers as in the PCR. LSU, *RPB1* and *TSR* were sequenced by Genomed S.A., Warsaw, Poland. Forward and reverse sequences were assembled using the unipro ugene 41.0 software [26]. Sequence data generated in this study are available in the GenBank database under accession numbers: OQ034234, OQ034235, OQ108496, OQ055159, OQ055160, OR244328, OR244329, PP319029, PP329305, PP319030, PP329306, PP329307.

### Phylogenetic Analysis

Preliminary phylogenetic analysis was performed to infer the placement of the new species within a genus. Sequences obtained in this study were combined with sequences of taxa present in the study by Walther et al. [10] retrieved from GenBank. Methods described below were used to infer a maximum-likelihood phylogenetic tree. Sequences used in the preliminary analysis are shown in Supplementary Table 1. The obtained phylogenetic tree visualized in iTOL [27] is available as Supplementary Fig. 1. Closely related taxa were chosen based on the obtained phylogenetic tree.

To perform further phylogenetic analysis the sequences obtained in this study were combined with reference sequences of the selected representatives of *Mucorinae* retrieved from GenBank (Supplementary Table 2) [8, 9, 11]. To obtain the best possible resolution of the tree, sequences of 6 molecular markers were retrieved: *CFS*, ITS, LSU, *MCM7*, *TSR* and *RPB1* [8]. Separate alignments for each molecular marker were aligned using mafft [28]. Alignments were trimmed with locally installed trimal [29] with the use of automated1 algorithm. Alignments are available in supplementary materials (Supplementary Material). Nucleotide substitution models were selected using modeltest-ng [30] for each molecular marker separately. Selected nucleotide substitution models derived from modeltest-ng for each molecular marker are summarized in Table 1. Maximum-likelihood phylogenetic trees (with 1000 bootstrap replicates) were inferred with raxml-ng [31] based on each of the alignments to assess if the phylogenetic information was congruent between the markers. Sequences from each strain were merged in R 4.3.0 [32] with a consequences script [33]. Based on concatenated sequences a phylogenetic tree was inferred using two methods—maximum-likelihood and Bayesian inference. Maximum-likelihood tree was inferred using raxml-ng [31] with 1000 bootstrap replicates and previously selected nucleotide substitution model for each partition (Table 1). Bayesian inference tree was inferred in MrBayes3.2.6 [34] on one million generations. For Bayesian inference tree calculation TPM1uf, TPM3uf, TIM2, TIM3, TrN substitution models were replaced with GTR. However, the information about invariant sites and gamma distribution were retained for each partition (Table 1). Additionally topological congruence was tested using concaterpillar1.7.2 [35]—a GTR nucleotide substitution model was applied. Phylogenetic tree was visualized in R 4.3.0 [32] with the use of ggtree [36], treeio [37], and castor [38] packages. The tree was artificially rooted with the type material sequences of *Mucor luteus* Linnemann ex Wrzosek and *Mucor racemosus* f. *racemosus* Fresenius. In order to calculate genetic distance between chosen ITS sequences the sequences (MH855663, MF615064, JN206273, JN206272, MK163846, OQ034234, OQ034235) were aligned using mafft algorithm [28], then trimmed manually. The percentage of nucleotide differences was calculated using a fragment of 590 characters in unipro ugene.

**Table 1:** Selected nucleotide substitution models derived from modeltest-ng for each molecular marker used

DNA region	Selected nucleotide substitution model for Maximum-likelihood analysis	Selected nucleotide substitution model for Bayesian inference analysis
ITS	TPM1uf + G4	GTR + G4
LSU	TrN + I + G4	GTR + I + G4
*CFS*	TrN + I + G4	GTR + I + G4
*MCM7*	TPM3uf + I + G4	GTR + I + G4
*RPB1*	TIM2 + G4	GTR + G4
*TSR*	TIM3 + G4	GTR + G4

## Results

Soil chemical composition is shown in Table 2. Limits of compounds for agricultural soil, specified by Polish law are shown as a reference [39]. The chemical analysis of soil samples showed high concentrations of hydrocarbons (mineral oil and aromatic hydrocarbons). High concentrations of some heavy metals (e.g., manganese) were also observed.

**Table 2:** Soil chemical composition

Compound	Concentration in soil (mg per kg of dry mass)	Limit for agricultural soil (mg per kg of dry mass)
*Heavy metals*		
Mercury (Hg) [mg/kg DM]	0.04	5
Chromium (Cr) [mg/kg DM]	9.2	500
Zinc (Zn) [mg/kg DM]	38	1000
Cadmium (Cd) [mg/kg DM]	0.2	5
Cobalt (Co) [mg/kg DM]	0.73	50
Copper (Cu) [mg/kg DM]	52	300
Molybdenum (Mo) [mg/kg DM]	0.32	50
Nickel (Ni) [mg/kg DM]	3.9	300
Lead (Pb) [mg/kg DM]	10	500
Aluminium (Al) [mg/kg DM]	530	Not specified
Iron (Fe) [mg/kg DM]	1100	Not specified
Manganese (Mn) [mg/kg DM]	1100	Not specified
Vanadium (V) [mg/kg DM]	2.7	Not specified
*Organic compounds*		
Gasoline total (C6–C12) [mg/kg DM]	149	1
Mineral oil (C12–C35) [mg/kg DM]	110	50
Sum of presented BTEX	0.96	Not specified
- Benzene [mg/kg DM]	< 0.02	0.1
- Ethylbenzene [mg/kg DM]	< 0.02	0.1
- Toluene [mg/kg DM]	0.02	0.1
- m-, p-, o- Xylene [mg/kg DM]	0.94	0.1
- Styrene [mg/kg DM]	< 0.02	0.1
Sum of presented PAH [mg/kg DM]	1.02	not specified
- Naphthalene [mg/kg DM]	0.012	0.1
- Anthracene [mg/kg DM]	0.046	0.2
- Chrysene [mg/kg DM]	0.229	0.2
- Benzo(a)anthracene [mg/kg DM]	0.079	0.1
- Dibenz(ah)anthracene [mg/kg DM]	< 0.005	not specified
- Benzo(a)pyrene [mg/kg DM]	0.081	0.1
- Benzo(b)fluoranthene [mg/kg DM]	0.192	0.1
- Benzo(k)fluoranthene [mg/kg DM]	0.189	0.1
- Benzo(ghi)perylene [mg/kg DM]	0.104	0.2
- Indeno(123-cd)pyrene [mg/kg DM]	0.085	0.2

The concaterpillar1.7.2 analysis [35] identified two datasets (ITS, *RPB1*, *TSR* and LSU, *MCM7*, *CFS*) which were incongruent with each other (*p* < 0.001). However, as the phylogenetic position of *Mucor thermorhizoides* sp. nov. on all trees based on single molecular markers was consistent, the data was concatenated (Supplementary Material 2).

**Fig. 2:**
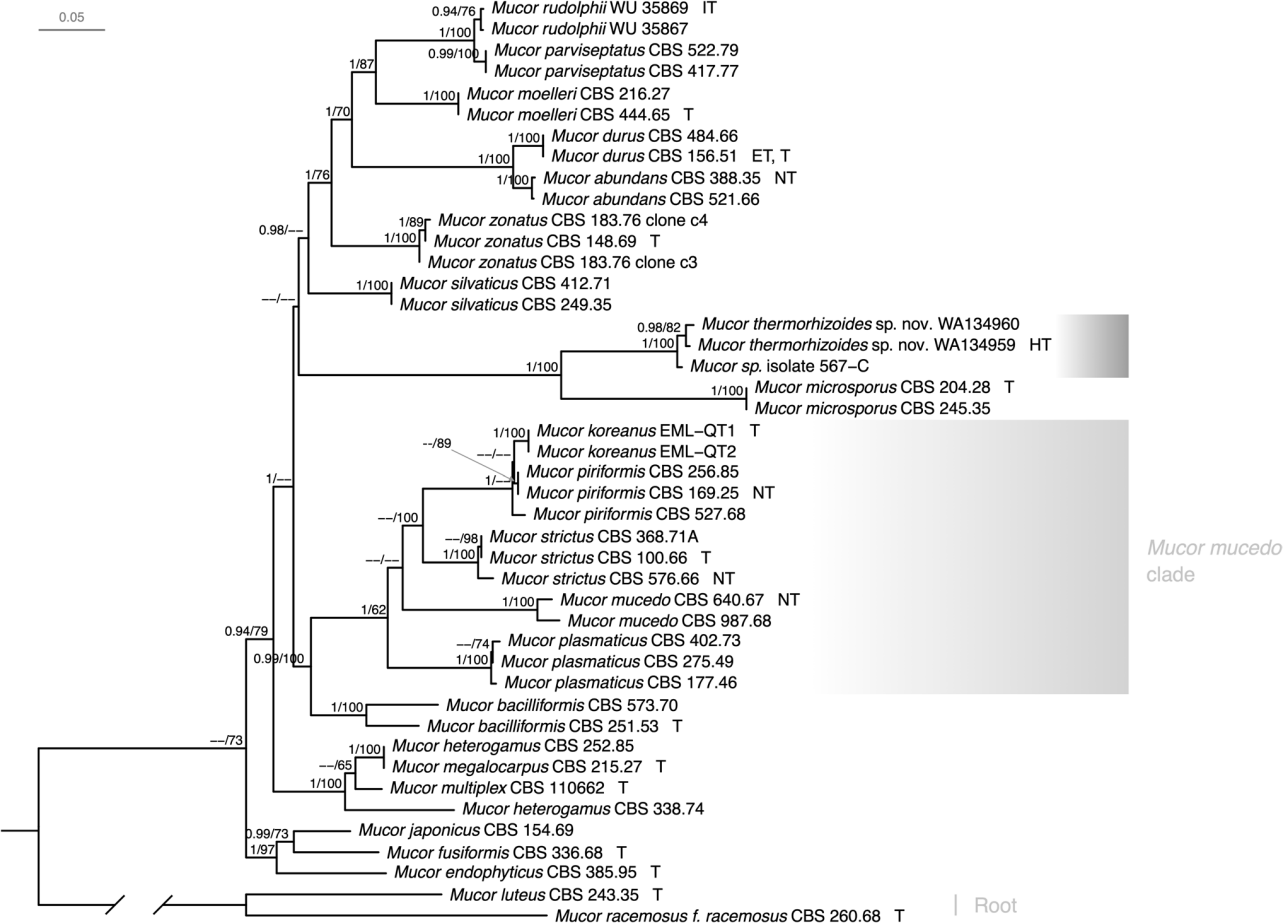
Maximum likelihood and Bayesian inference phylogram of selected *Mucor* species. The phylogram was inferred from 44 strains and 4001 characters (1–587 bp *TSR*, 588–1440 bp *RPB1*, 1441–1995 bp ITS, 1996–2687 bp LSU, 2688–3269 bp *MCM7*, 3270–4001 bp *CFS*) based on partitioned data analysis. Posterior probabilities (≥ 0.9) and maximum-likelihood bootstrap support values (≥ 60%) are indicated above the branches. The tree is rooted using sequences of *Mucor luteus* (CBS 243.35) and *Mucor racemosus* (CBS 260.68). The new species is highlighted with dark grey. *Mucor mucedo* clade is highlighted with light-grey. Types, ex-neotypes, ex-isotypes, ex-types, and ex-holotypes are denoted by T, NT, IT, ET, and HT respectively. More specific information on typification can be found in Supplementary Table 2

Maximum likelihood analysis and Bayesian inference analysis resulted in trees with the same topology. The general tree topology is in accordance with the phylogenies published by Walther et al. [9, 10]. Sequences of *Mucor thermorhizoides* sp. nov. obtained in this study formed a distinct, well-supported, monophyletic clade together with the unidentified *Mucor* sp. isolate 567-C [17] sister to *Mucor microsporus* Namysl. (Fig. 2). Genetic distance in ITS sequence between *M. thermorhizoides* and *M. microsporus* exceeds 10%.

## Taxonomy

**Fig. 3:**
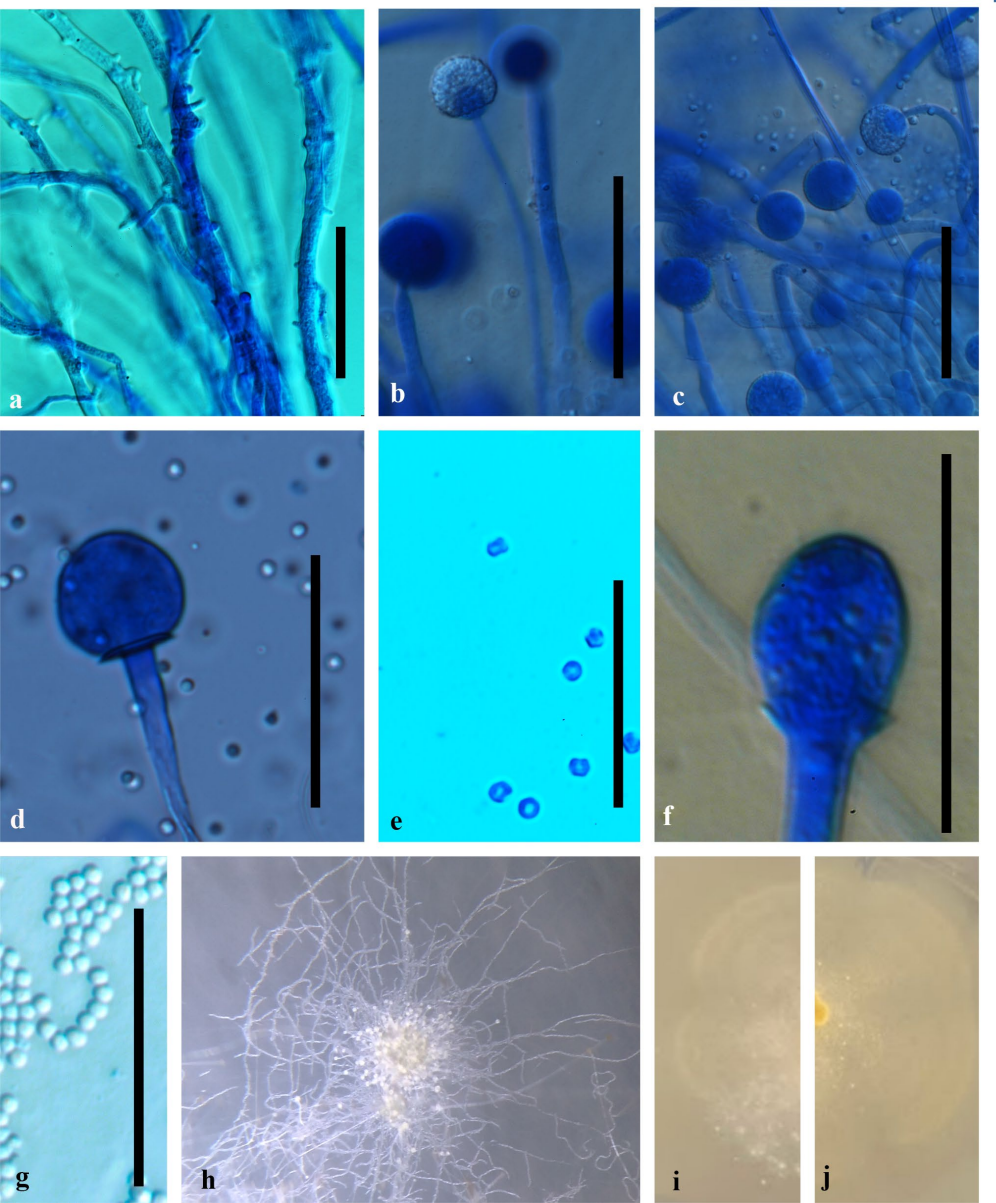
Morphology of *Mucor thermorhizoides* WA134959 **a** hyphae with aerial rhizoids; **b** and **c** sporangia of on shorter sporangiophores with spinulose walls when young; **d** columellae of sporangia from lower layer; **e** sporangiospores from lower layer sporangiophores; ** f** columellae of sporangia from upper layer; ** g** sporangiospores from upper layer sporangiophores; **h** cluster of sporangiophores under dissecting microscope; **i** colony averse on MEA medium; **j** colony reverse on MEA medium. Scale bar: 50 μm

*Mucor thermorhizoides* Abramczyk, sp. nov. (Fig. 3).

MycoBank: MB847498.

*Typification:* POLAND, LOWER SILESIA, MARCISZÓW COUNTY: Wieściszowice, Kolorowe Jeziorka preserved nature post-mining area, path from Purpurowe Jeziorko to Niebieskie Jeziorko (50°49.512’N, 15°58.332’E, WGS84), forest soil, 25 Jun 2018, *B. Abramczyk* (**holotype** WA134959). Ex-type culture in CBS-KNAW culture collection: CBS 149760. GenBank: OQ034234 (ITS), OR244328 (LSU), OQ055159 (*CFS*), OQ108496 (*MCM7*), PP329305 (*RPB1*), PP329306 (*TSR*).

*Etymology*: The specific epithet refers to extensive rhizoid formation in elevated temperatures.

*Diagnosis: Mucor thermorhizoides* is morphologically characterized by the extensive formation of rhizoids in elevated temperatures. It produces two layers of yellowish sporangia, the ones on shorter sporangiophores are 46 ± 9 μm diameter (30 measurements) and produce angular spores, whilst the ones on longer sporangiophores are 26 ± 4 μm diameter (35 measurements) and produce globular spores.

*Description:* Mycelium non-septate, hyaline, 5 μm diameter, may form synnema, with abundant rhizoids also on aerial hyphae, especially in temperatures above 25 °C. Sporangiophores simple, non-septated, smooth-walled, hyaline, and organized in two layers. Shorter sporangiophores are swollen, approx. 0.15–0.25 mm long (30 measurements), and longer sporangiophores are thinner, approx. 0.5–1.5 mm long (35 measurements). Sporangia of both types yellowish, with well visible lipid droplets, as defined by Koch et al. [40]. Sporangia on shorter sporangiophores 46 ± 9 μm diameter (30 measurements), with spinulose walls when young, with ovoid, smooth columellae, and produce angular spores of 4.6 ± 1 μm diameter (62 measurements). Sporangia on longer sporangiophores 26 ± 4 μm diameter (35 measurements), smooth-walled, globular, smooth columellae, produce globular spores of 5 ± 1 μm diameter (30 measurements). Spores smooth-walled and hyaline in both types. Chlamydospores and zygospores were not observed.

*Culture characteristics.* Colonies were examined on MEA medium one week post inoculation. At 4 °C colonies are flat and white in colour. At 23 °C they are cottony, Pale Chalcedony Yellow to Strontian Yellow, centres are more intense in colour on the reverse, margins are irregular. At 25 °C white, hyphae bent, dense, and felted, with clusters of whitish-yellowish sporangia.

**Fig. 4:**
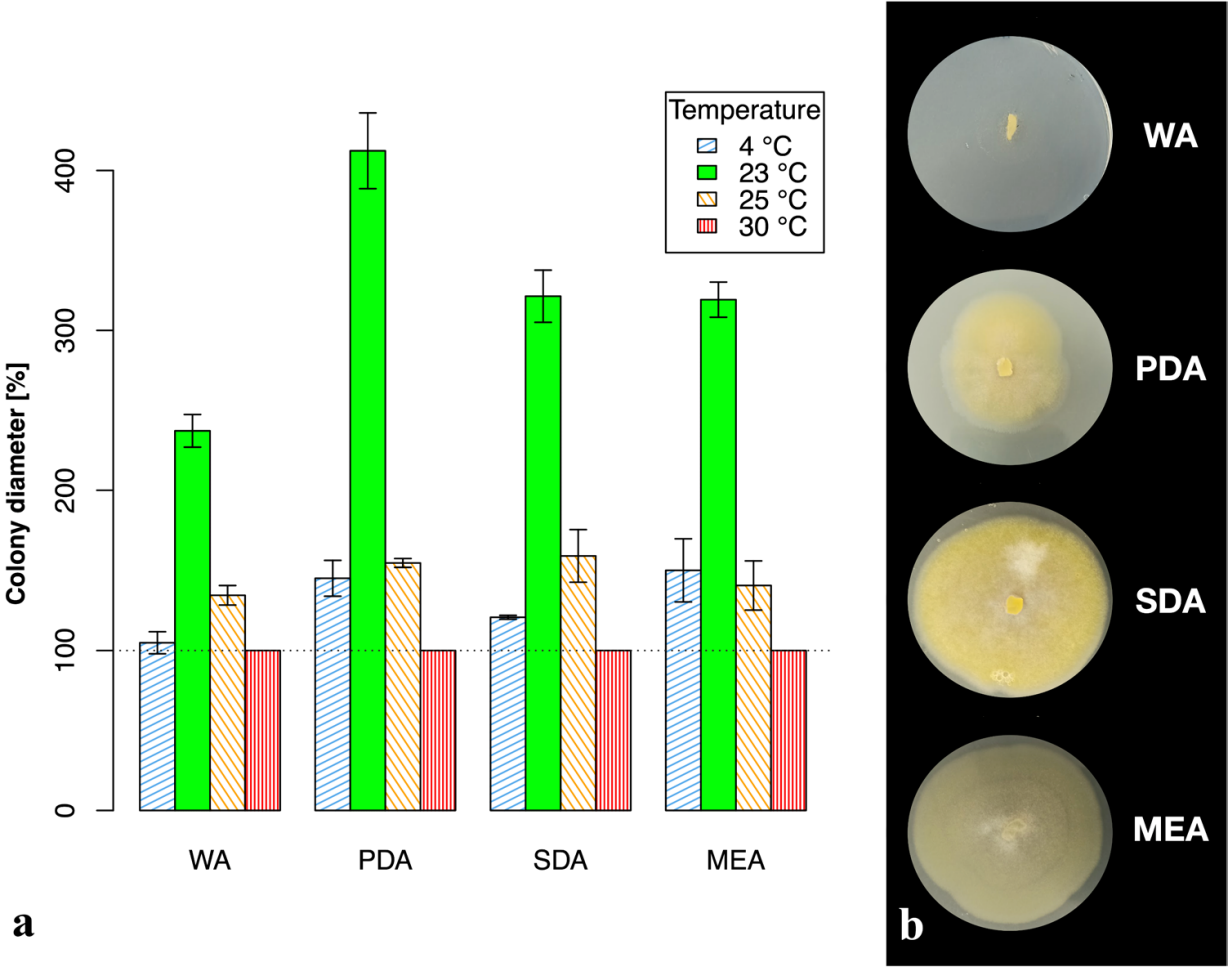
Morphology and growth of *Mucor thermorhizoides* on different media **a** increase of mean colony diameter in % during colony incubation in three different temperatures on WA, PDA, SDA and MEA media (bars represent mean ± SD); **b** morphology of *Mucor thermorhizoides* colonies on WA, PDA, SDA, and MEA media at 23 °C

Morphological characteristics of *Mucor* colonies on different media are shown in Fig. 4**.** Colonies on WA flat and hyaline. On PDA cottony, Citron Yellow. On SDA leather-like colonies, Honey Yellow, reverse Strontian Yellow.

**Fig. 5:**
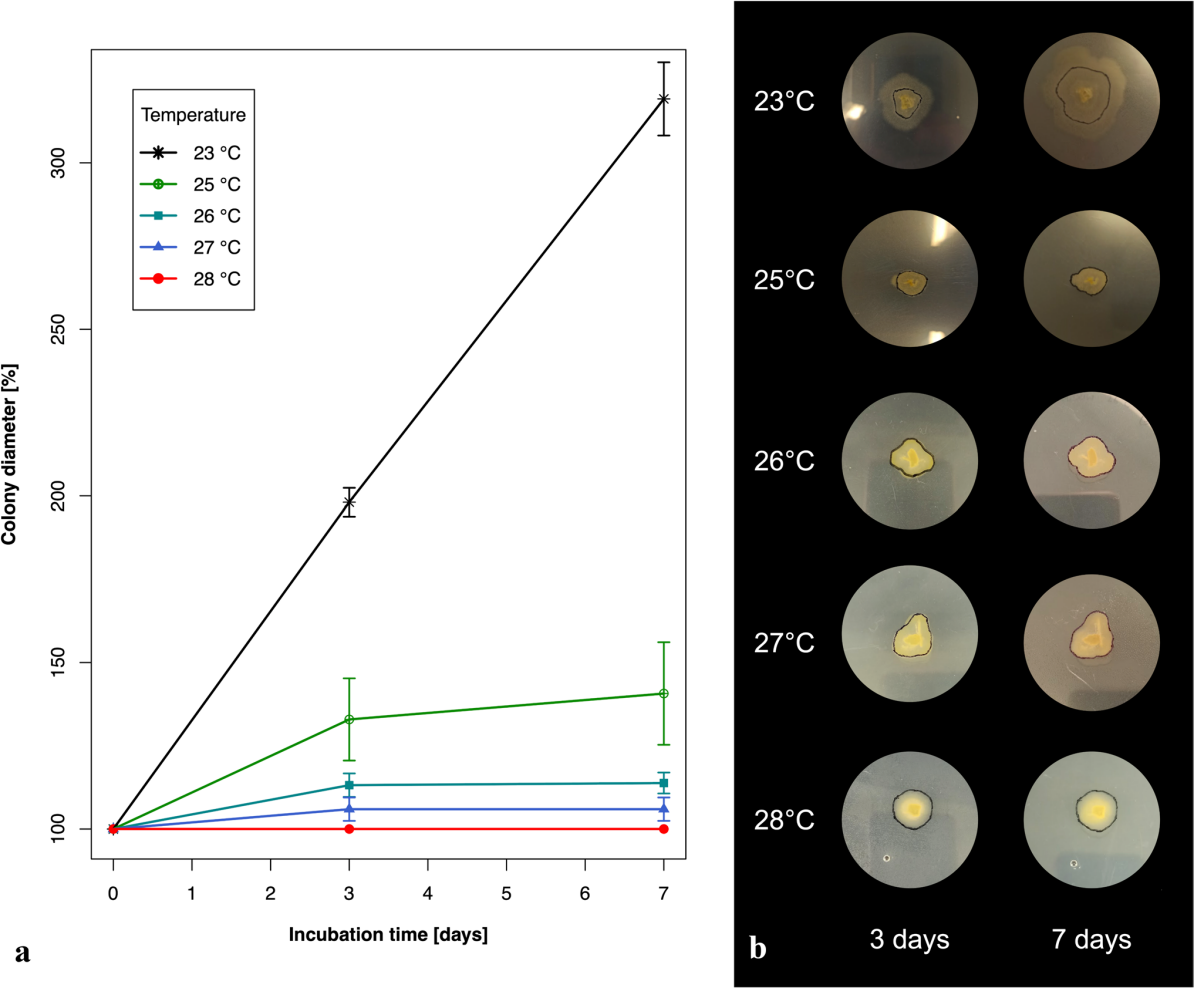
The maximum growth temperature of *Mucor thermorhizoides* **a** increase of mean colony diameter in % during colony incubation in 5 different temperatures (bars represent mean ± SD); **b** colony growth in 5 different temperatures

*Physiology.* Growth of colonies was examined at 4 different temperatures: 4, 23, 25, and 30 °C, during 7 days. Diameter of colonies on MEA at 4 °C increased by ca. 7 mm, at 23 °C ca. 32 mm, at 25 °C ca. 6 mm, and no growth was observed at 30 °C. The maximum growth temperature test was performed on MEA and it was determined as 27 °C (Fig. 5).

*Specimens examined:* CBS 149760 (= WA134959), WA134960.

*Habitat:* Forest soil or wooden artefacts.

*Distribution:* Poland and Greenland.

## Discussion

It is generally recommended to study multiple specimens of fungi in order to describe a new species [41]. However, for some rare taxa which lack a formal description, it may slow down further understanding of their ecological role, estimation of their actual distribution, and extinction risk assessments. We believe that here-described *Mucor thermorhizoides* represents this kind of rare mucoralean species. None of the multiple attempts to reisolate the species from the same location yielded expected results, i.e., other strains of *M. thermorhizoides*. The representatives of the genus *Mucor* are generally considered common and ubiquitous soil saprotrophs. However, the genus encompasses also rare and undersampled ones. Formal description of *Mucor thermorhizoides*, which, we believe, is an example of such a rare taxon, will accelerate obtaining knowledge on its occurrence, and, consequently, on its ecology and biogeography.

In our phylogenetic analyses, sequences belonging to *Mucor thermorhizoides* form a distinct, well-supported clade sister to *Mucor microsporus*. However, given the paraphyly of the genus [9] and considerable phylogenetic distance of the newly described species from the type species *M. mucedo*, its taxonomic placement is likely to change once the genus is thoroughly revised.

Genetic distance analysis of the ITS region also supported the delimitation of *M. thermorhizoides* as separate new species. The percentage of nucleotide difference in the ITS region to the sister *M. microsporus* is 14–16% and it highly exceeds the thresholds calculated for other *Mucor* species, i.e., 0.2–5.3% [9]. Therefore, additional phylogenetic markers were used to confirm its phylogenetic placement close to *M. microsporus.*

The name *Mucor microsporus* was first used by Bonorden [42]. However, the original species description is very vague. Further, the same name was introduced in an illegitimate way three more times as *M. microsporus* Namysl. [43], *M. microsporus* Naumov [44], and *M. microsporus* (Tiegh.) Mig. [45]. The third one refers to *Rhizopus microsporus* Tiegh. which currently does not belong to the *Mucor* genus. However, the *M. microsporus* Namysl. and *M. microsporus* Naumov were misinterpreted for a long time. Finally, Schipper [5] selected *M. microsporus* Namysl. over *M. microsporus* Naumov to characterize the species. The Author synonymized *Mucor cylindrosporus* Y. Ling with *M. microsporus* Namysl, using strain CBS 204.28 to characterize and describe *M. microsporus* Namysl in detail. All later phylogenetic studies, including this one, use this material as an ex-type for *M. microsporus.*

In his original description of *M. microsporus* Namysłowski [43] reported that spores are hyaline, ellipsoid and small, i.e., 2–3 µm × 1.5 µm. Further, Schipper [5] reports that in strain CBS 204.28 the sporangiospores are cylindrical to ellipsoidal, 3.4–5.4 × 2–3 µm. The spores of *M. thermorhizoides* differ from the spores of *M. microsporus* in shape and size, globular spores of *M. thermorhizoides* have 4.8 ± 1.2 μm diam, and angular spores have 4.6 ± 1 μm diam. Although Namysłowski [43] described simple sporangiophores up to 20 mm in height, Schipper [5] reported that usually they are much shorter, i.e., 3–4 mm in height, and only occasionally up to 23 mm. *M. thermorhizoides* has two types of sporangiophores, shorter up to 3 mm in height and longer up to 15 mm. Although sporangia of *M. thermorhizoides* are yellowish like in the case of *M. microsporus*, they are smaller, i.e., up to 50 μm in diameter, whilst both Namysłowski [43] and Schipper [5] were describing sporangia up to 80 μm in diameter. Most importantly, *M. thermorhizoides* forms extensive rhizoids, especially in higher temperatures, which is its unique characteristic not observed in any of the closely related *Mucor* species. The rhizoid formation was for a long time considered diagnostic for *Rhizopus* and *Rhizomucor* genera (e.g., [5, 7]). After the reclassification of some mesophilic *Rhizomucor* species, the potential to form rhizoids is plesiomorphic in *Mucorales* [9]*,* as the formation of rhizoids was also observed in *M. endophyticus* (R.Y. Zheng and H. Jiang) J. Pawłowska and G. Walther and *M. irregularis* Stchigel, Cano, Guarro and Ed. Álvarez.

The UNITE species hypothesis (SH [15]) matching at 1.5% threshold clusters newly described *M. thermorhizoides* with SH1102029.09FU. Although the 1.5% ITS sequence dissimilarity threshold is relatively high for fungi in general, it fits within the thresholds calculated for other *Mucor* species, i.e., 0.2–5.3% [9]. The observed mismatches between sequences are mostly located in the most variable region of ITS, i.e., ITS2. Although potentially they may result from few sequencing errors, taking into account their position we believe that they reflect real variability within this clade. In our phylogenetic analysis (see Fig. 2) the isolate from Greenland forms a well-supported clade with the WA134959 isolate described here. However, in order to verify whether both isolates represent the same species, morphological studies and mating experiments are necessary. Isolating more strains representing this clade will also help to answer this question.

Physiological data obtained show that *M. thermorhizoides* has a similar range of temperature tolerance as *M. microsporus*. Even though maximum growth temperature was not defined for the latter, both species are able to grow from 4 to 5 °C up to 25 °C and there is no noticeable growth in 30 °C [5]. Growth tests performed in this study show that *M. thermorhizoides* has an atypically slow growth rate for *Mucor* species. Radial growth of its colonies at 23 °C reaches only ca. 2.3 mm per day whilst for most *Mucor* species it exceeds 6 mm per day at optimal growth temperature (for *M. microsporus* 6.1 mm per day at 20 °C) [5, 46]. However, taking into account the ability of *M. thermorhizoides* to grow at 4 °C, the temperature of soil at the moment of sampling (2 °C), and the fact that sequences forming matching species hypothesis come only from Greenland, we assume that this species is psychrotolerant. Studies performed on other mucoralean psychrotolerant species *Mucor strictus* Hagem showed a severe decrease of growth rate just several degrees Celsius over the optimal growth temperature (tests performed on solid media) [47].

Finally, *M. thermorhizoides* was isolated from soil with elevated concentrations of molybdenum, benzene, naphthalene, and mineral oils (see Table 2 for details) in the pyrite post-mining area in Rudawy Janowickie Mountains. Interestingly, *M. microsporus* described by Namysłowski [43] was also isolated from mountain soil in Central Europe, from Czarnohora (currently western Ukraine). However, unlike *M. thermorhizoides, M. microsporus* was isolated from pristine montane meadow habitat, locally called ‘polonyna’. These differences may suggest that *M. thermorhizoides* is rather associated with anthropogenic habitats, whilst *M. microsporus* is typical for natural ones. However the occurrence data for both taxa are limited and therefore these claims should be treated with caution.

According to the UNITE database *M. microsporus* (SH1102032.09FU at 1.5% threshold) represents European species that was detected in eDNA extracted from soil samples from Sweden, Estonia, and Czech Republic. The known strains of this species originate from France and Austria. Interestingly, there are also some other species hypotheses known from environmental soil sampling which seem to be closely related to the *M. microsporus* and *M. thermorhizoides* group. These SH1102030.09FU, SH1102038.09FU, SH1102037.09FU, and SH1102031.09FU were detected in DNA from soil in Estonia, Lithuania, Sweden, Norway, Switzerland, Spain, and the USA. However, they are not linked to any described *Mucor* species so far. This indicates a poor state of knowledge of this particular *Mucor* group that represents understudied and highly variable lineage of this genus.

## Conclusion

Our study proves that uncovering *Mucor* taxa known previously only as DNA based species hypothesis is feasible. Here a new mucoralean species *Mucor thermorhizoides* was described basing on an isolate from Poland. *Mucor microsporus* is the most closely related to *Mucor thermorhizoides* from all known *Mucor* species. However eDNA data suggest that together they represent a speciose understudied mucoralean lineage, for which further research is needed to better understand its ecology and evolutionary history.

### Supplementary Information

Below is the link to the electronic supplementary material.

Supplementary Figure 1 (PDF 98 KB)

Supplementary Table 1 (XLSX 15 KB)

Supplementary Table 2 (XLSX 12 KB)

Supplementary Material 1 (ZIP 16 KB)

Supplementary Material 2 (ZIP 4 KB)

Supplementary files legends (DOCX 9 KB)

## Data Availability

All nucleotide sequences generated in this study are available in the GenBank database under following accession numbers: OQ034234, OQ034235, OQ108496, OQ055159, OQ055160, OR244328, OR244329, PP319029, PP329305, PP319030, PP329306, PP329307. Sequence alignments used for calculating the presented phylogenetic tree are available in supplementary materials. Specific physiological data obtained in this study (growth tests) can be obtained by e-mail request to the corresponding author. Ex-type isolate of *Mucor thermorhizoides* sp. nov. Abramczyk can be obtained from CBS-KNAW fungal collection, where it was deposited (CBS 149760). Type material is available in the Herbarium of University of Warsaw (WA134959).
