# Supplementary figures and images for: Mucor thermorhizoides—A New Species from Post-mining Site in Sudety Mountains (Poland)
Source: Curr Microbiol. 2024 Jun 1;81(7):201. doi: 10.1007/s00284-024-03708-7 (PMC11144139; doi:10.1007/s00284-024-03708-7)

Tree scale: 0.1

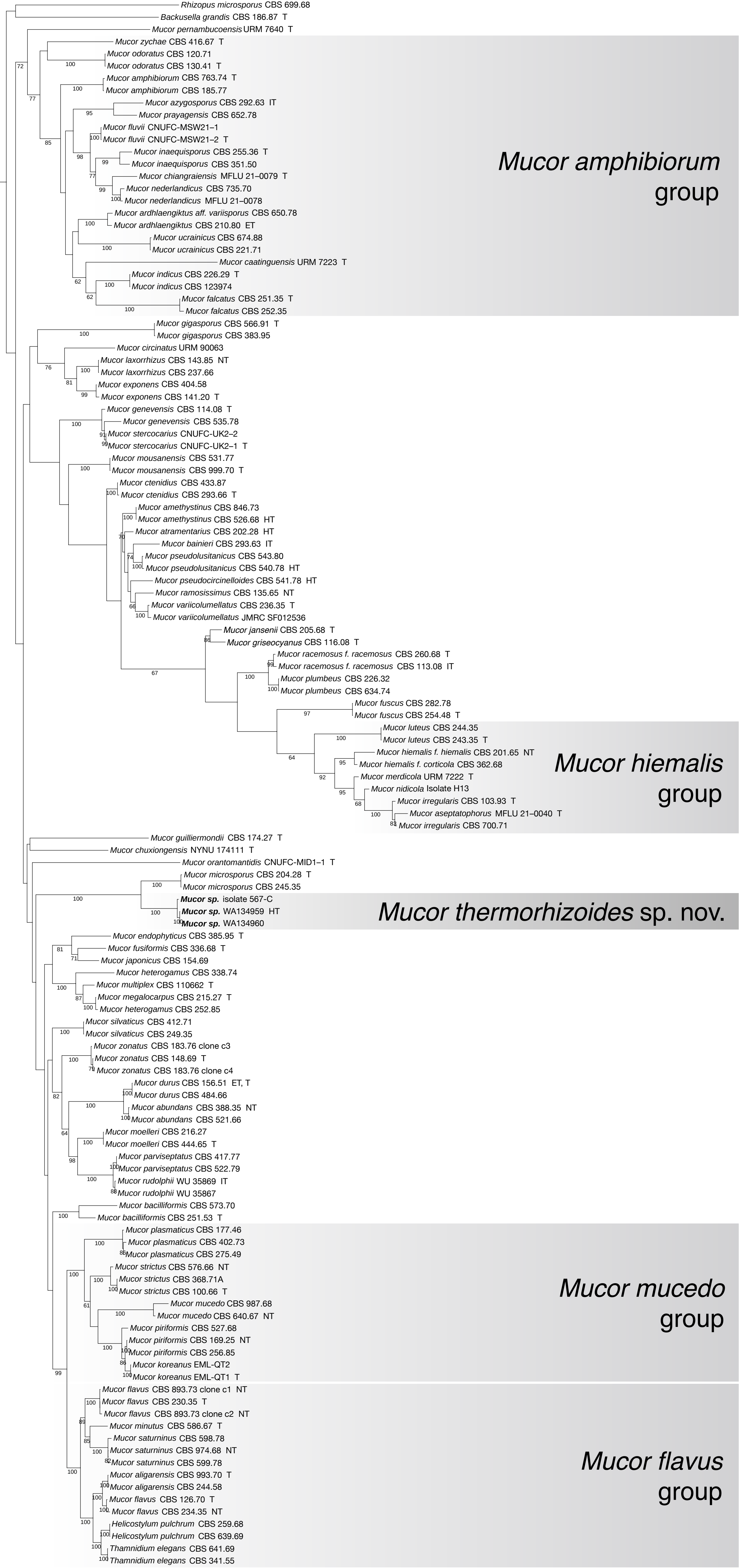

Supplement: Supplementary file 1 — Supplementary Figure 1 (PDF 98 KB) [file 284_2024_3708_MOESM1_ESM.pdf]
