# Supplementary material for: Mucor thermorhizoides—A New Species from Post-mining Site in Sudety Mountains (Poland)
Source: Curr Microbiol. 2024 Jun 1;81(7):201. doi: 10.1007/s00284-024-03708-7 (PMC11144139; doi:10.1007/s00284-024-03708-7)
Supplement: Supplementary file 6 — Supplementary files legends (DOCX 9 KB) [file 284_2024_3708_MOESM6_ESM.docx]

**LEGENDS FOR SUPPLEMENTARY MATERIALS**

**Supplementary Figure 1.** Maximum likelihood and Bayesian inference phylogram of selected *Mucor* species. The phylogram was inferred from 128 strains and 4212 characters based on partitioned data analysis. Bootstrap support values (≥ 60%) are indicated above the branches. The tree is rooted using sequences of *Rhizopus microsporus* (CBS 699.68) and *Backusella grandis* (CBS 186.87). The new species is highlighted with dark-grey. Main *Mucor* clades are highlighted with light-grey. Types, ex-neotypes, ex-isotypes, ex-types, and ex-holotypes are denoted by T, NT, IT, ET, and HT, respectively. More specific information on typification can be found in Supplementary Table 1.

**Supplementary table 1.** Data used for preliminary phylogenetic analysis in this study with corresponding GenBank accession numbers. Types, ex-neotypes, ex-types, ex-holotypes and ex-isotypes are denoted by T, NT, ET, HT, and IT respectively. Sequences obtained in this study are shown in bold red letters. Sequences used to root the tree are shown in bold black letters. Strain status information was derived from CBS database (<https://wi.knaw.nl/page/fungal_table> accessed 5.01.2023), unless stated otherwise in the status reference column[11, 48, 49, 50, 51, 52]*.*

**Supplementary table 2.** Data used for phylogenetic analysis in this study with corresponding GenBank accession numbers. Types, ex-neotypes, ex-types, ex-holotypes and ex-isotypes are denoted by T, NT, ET, HT, and IT respectively. Sequences derived in this study are shown in bold red letters. Sequences used to root the tree are shown in bold black letters. Strain status information was derived from CBS database (<https://wi.knaw.nl/page/fungal_table> accessed 5.01.2023), unless stated otherwise in the status reference column [11, 52]*.*

**SUPPLEMENTARY MATERIAL 1** (as compressed zip):

**cfs_Mucor_trim.** *CFS* gene sequences aligned with Mafft [28] and trimmed using Trimal [29], automated1 algorithm.

**ITS_Mucor_trim.** *ITS* sequences aligned with Mafft [28] and trimmed using Trimal [29], automated1 algorithm.

**LSU_Mucor_trim.** *LSU* sequences aligned with Mafft [28] and trimmed using Trimal [29], automated1 algorithm.

**mcm_Mucor_trim.** *MCM7* gene sequences aligned with Mafft [28] and trimmed using Trimal [29], automated1 algorithm.

**rpb_Mucor_trim.** *RPB1* gene sequences aligned with Mafft [28] and trimmed using Trimal [29], automated1 algorithm.

**tsr_Mucor_trim.** *TSR* gene sequences aligned with Mafft [28] and trimmed using Trimal [29], automated1 algorithm.

**SUPPLEMENTARY MATERIAL 2** (as compressed zip):

**cfs_tree.** Maximum likelihood phylogenetic tree inferred based on aligned *CFS* marker sequences with the use of Raxml-NG [31].

**ITS_tree**. Maximum likelihood phylogenetic tree inferred based on aligned ITS marker sequences with the use of Raxml-NG [31].

**LSU_tree.** Maximum likelihood phylogenetic tree inferred based on aligned LSU marker sequences with the use of Raxml-NG [31].

**Mcm7_tree.** Maximum likelihood phylogenetic tree inferred based on aligned *MCM7* marker sequences with the use of Raxml-NG [31].

**rpb1_tree.** Maximum likelihood phylogenetic tree inferred based on aligned *RPB1* marker sequences with the use of Raxml-NG [31].

**tsr_tree.** Maximum likelihood phylogenetic tree inferred based on aligned *TSR* marker sequences with the use of Raxml-NG [31].
